# Prenatal Exposure to Persistent Organochlorines and Childhood Obesity in the U.S. Collaborative Perinatal Project

**DOI:** 10.1289/ehp.1205901

**Published:** 2013-06-25

**Authors:** Lea A. Cupul-Uicab, Mark A. Klebanoff, John W. Brock, Matthew P. Longnecker

**Affiliations:** 1 Epidemiology Branch, National Institute of Environmental Health Sciences, National Institutes of Health, Department of Health and Human Services, Research Triangle Park, North Carolina, USA; 2 Center for Population Health Research, National Institute of Public Health, Cuernavaca, Morelos, Mexico; 3 The Ohio State University College of Medicine, Columbus, Ohio, USA;; 4 The Research Institute at Nationwide Children’s Hospital, Columbus, Ohio, USA; 5 National Center for Environmental Health, Centers for Disease Control and Prevention, Atlanta, Georgia, USA

## Abstract

Background: In some previous studies, prenatal exposure to persistent organochlorines such as 1,1,-dichloro-2,2-bis(*p*-chlorophenyl)ethylene (*p,p´*-DDE), polychlorinated biphenyls (PCBs), and hexachlorobenzene (HCB) has been associated with higher body mass index (BMI) in children.

Objective: Our goal was to evaluate the association of maternal serum levels of β-hexachlorocyclohexane (β-HCH), *p,p´*-DDE, dichlorodiphenyltrichloroethane (*p,p´*-DDT), dieldrin, heptachlor epoxide, HCB, *trans*-nonachlor, oxychlordane, and PCBs with offspring obesity during childhood.

Methods: The analysis was based on a subsample of 1,915 children followed until 7 years of age as part of the U.S. Collaborative Perinatal Project (CPP). The CPP enrolled pregnant women in 1959–1965; exposure levels were measured in third-trimester maternal serum that was collected before these organochlorines were banned in the United States. Childhood overweight and obesity were defined using age- and sex-specific cut points for BMI as recommended by the International Obesity Task Force.

Results: Adjusted results did not show clear evidence for an association between organochlorine exposure and obesity; however, a suggestive finding emerged for dieldrin. Compared with those in the lowest quintile (dieldrin, < 0.57 μg/L), odds of obesity were 3.6 (95% CI: 1.3, 10.5) for the fourth and 2.3 (95% CI: 0.8, 7.1) for the highest quintile. Overweight and BMI were unrelated to organochlorine exposure.

Conclusions: In this population with relatively high levels of exposure to organochlorines, no clear associations with obesity or BMI emerged.

Citation: Cupul-Uicab LA, Klebanoff MA, Brock JW, Longnecker MP. 2013. Prenatal exposure to persistent organochlorines and childhood obesity in the U.S. Collaborative Perinatal Project. Environ Health Perspect 121:1103–1109; http://dx.doi.org/10.1289/ehp.1205901

## Introduction

Childhood obesity is of public health concern worldwide (Lobstein et al. 2004). In the United States, the prevalence of obesity among children 6–11 years of age increased from 4% in 1971–1974 to 20% in 2007–2008 (Orsi et al. 2011). Obesity during childhood is predictive of obesity in adulthood (Orsi et al. 2011). Similar to obese adults, children who are obese are at increased risk of developing adverse health conditions such as insulin resistance, type 2 diabetes, hypertension, dyslipidemia, and asthma (Lobstein et al. 2004). Excess energy intake and low levels of physical activity are well documented risk factors for childhood obesity (Lobstein et al. 2004). The role of an adverse fetal environment (e.g., maternal diabetes, malnutrition, smoking) in the programming of childhood obesity is supported by previous studies (Huang et al. 2007; Oken and Gillman 2003). Emerging literature from animal models also suggests that prenatal exposure to endocrine-disrupting chemicals (e.g., tributyltin) might predispose the exposed subjects to greater accumulation of body fat later in life (Grun and Blumberg 2009).

Organochlorine compounds are manufactured chemicals that were widely used as pesticides [e.g., DDT (dichlorodiphenyltrichloroethane), aldrin, dieldrin, HCB (hexachlorobenzene)] and in industrial processes [PCBs (polychlorinated biphenyls)] between the 1950s and 1980s; they are ubiquitous in the environment, tend to bioaccumulate, and have a high affinity for fatty tissues [Centers for Disease Control and Prevention (CDC) 2009]. Organochlorines at high levels are known to be toxic for wildlife and humans. The use of these chemicals is presently banned or restricted; but because of the chemicals’ persistence in the environment, adverse health outcomes related to background levels of exposure are still a concern for the general population (World Health Organization 2010). In humans, prenatal exposure to some persistent organochlorines such as *p,p´*-DDE [1,1-dichloro-2,2-bis(*p*-chlorophenyl)ethylene, the main breakdown product of DDT], PCBs, and HCB has been associated with higher body weight or body mass index (BMI; kilograms per meter squared) across a wide range of ages; however, overall the results from previous studies are equivocal (Tang-Peronard et al. 2011).

The main purpose of the present study was to assess the association between prenatal exposure to persistent organochlorines [i.e., β-hexachlorocyclohexane (β-HCH), *p,p´*-DDE, *p,p´*-DDT, dieldrin, heptachlor epoxide, hexachlorobenzene, *trans*-nonachlor, oxychlordane, and PCBs] and subsequent childhood obesity in a relatively large population whose exposure levels were measured in maternal serum collected before these chemicals were banned in the United States. Potential interactions reported by earlier studies (e.g., *p,p´*-DDE and maternal smoking) were explored using child’s BMI as the outcome. We also evaluated whether any associations appeared to differ according to size at birth, which might mediate the relationship between prenatal exposures and childhood body weight.

## Methods

The present study was based on the U.S. Collaborative Perinatal Project (CPP). Detailed information about the CPP is available elsewhere (Niswander and Gordon 1972). Briefly, the CPP was a prospective cohort that enrolled pregnant women from 1959 to 1965 at 12 study centers across the United States. Women provided nonfasting blood samples throughout their pregnancy and at delivery; serum was stored in glass at –20°C with no recorded thaws. The CPP enrolled about 42,000 women who delivered 55,000 babies. These children were systematically assessed for various outcomes until 7 years of age; the follow-up rate at this age was about 75%. Exposure to organochlorines was measured in a subset of the CPP cohort; children were eligible if they were singleton and live-born, and there was a third trimester maternal serum sample of 3 mL available. Of the 44,075 children who met the eligibility criteria, 1,200 were selected at random and 1,623 were selected according to sex-specific birth defects or performance on various neurodevelopmental tests (Longnecker et al. 2001). The sampling was done independently, and the 71 children selected for more than one subgroup were included in the analysis only once. The study complied with all applicable requirements of the U.S. regulations to conduct research in humans, women gave oral informed consent prior to the (CPP) study, and the Institutional Review Board of the National Institute of Environmental Health Sciences deemed that the present analysis was exempt.

*Organochlorine exposure.* Maternal serum samples were analyzed for β-HCH, *p,p´*-DDE, *p,p´*-DDT, dieldrin, heptachlor epoxide, HCB, *trans*-nonachlor, oxychlordane, and 11 PCB congeners (28, 52, 74, 105, 118, 138, 153, 170, 180, 194, and 203) at the CDC from 1997 to 1999. Quantification of these organochlorines was done using electron capture detection after solid-phase extraction, cleanup, and dual-column gas chromatography (Brock et al. 1996). Measured values reported by the laboratory that were below the limit of detection (LOD) were used in the analyses; no imputation of values below the LOD was done (Longnecker et al. 2002). The average proportion of chemicals recovered by extraction ranged between 45 and 80; for most the recovery was > 60% (i.e., recovery was < 60% for PCB congeners 170, 194, and 203) (Brock et al. 1996). The results are shown without recovery adjustment (Longnecker et al. 2002). For the present analysis, the concentrations of the 11 PCB congeners were summed to calculate total PCBs. Serum triglycerides and total cholesterol (milligrams per deciliter) were measured with standard enzymatic methods (Longnecker et al. 2002).

Organochlorines were measured in third- rather than first-trimester maternal serum, which is potentially the critical period for developmental exposure. However, previous data from the CPP have shown that serum levels of persistent organochlorines (i.e., *p,p´*-DDE and PCBs) early in pregnancy correlated well with those found at the end of pregnancy (Longnecker et al. 1999).

*Outcomes.* At the 7-year examination, children had their height and weight measured by trained personnel as part of the CPP study. Childhood overweight and obesity were defined using age- and sex-specific cut points for BMI as recommended by the International Obesity Task Force (IOTF), which allows prevalence comparisons across countries (Cole et al. 2000). These cut points correspond to a BMI approximately above the 90th centile for overweight and approximately above the 97th centile for obesity (specific cut points defined by 6-month age bands for girls and boys, and ranged from 17.53 to 18.44 for overweight and from 20.08 to 21.60 for obesity) (Cole et al. 2000). The IOTF cut points are considered equivalent to the well-established cut points for BMI to define overweight (25 kg/m^2^) and obesity (30 kg/m^2^) in adults (Cole et al. 2000). The primary outcome for the present analysis was childhood obesity. Because the number of children classified as obese was small, overweight including obesity (hereafter referred to as overweight unless otherwise noted) and BMI as a continuous variable were also modeled as secondary outcomes.

*Covariates.* Socioeconomic index was defined based on education level and occupation of the head of the household and the family income at enrollment (Longnecker et al. 2002). Maternal smoking during pregnancy was ascertained at enrollment. Pregnancy weight gain (kilograms) was calculated from self-reported prepregnancy weight and weight measured at the end of pregnancy. Maternal prepregnancy BMI was calculated from self-reported prepregnancy weight and measured height. Information about breastfeeding was recorded in the nursery wards and refers to whether the child was breastfed during the first week of life.

Organochlorine levels were missing for 3% of the 2,823 children selected, mostly because the measured value did not meet the quality control standards for acceptance (Brock et al. 1996). The present analysis was restricted to children whose organochlorine exposure was measured and those with available height, weight, and exact age for the 7-year examination (*n* = 2,094). Children with implausible values for height or weight (*n* = 13) and those missing data on maternal prepregnancy BMI (*n* = 144), smoking (*n* = 11), pregnancy weight gain (*n* = 8), or birth order (*n* = 2) were excluded, leaving 1,916 children with data for analysis. The number of children included in analyses of each organochlorine ranged between 1,807 and 1,915 because of varying numbers of missing values for each of them.

*Statistical analysis.* The association of prenatal exposure to organochlorines with childhood obesity and childhood overweight was modeled with logistic regression. Separate models were fitted for each of the nine chemicals and each outcome. The shape of the relationships between the outcomes and exposures was examined using restricted cubic splines (of the exposure) with three knots (Harrell 2001), a method for modeling nonlinear relationships. In the spline models, odds ratios (ORs) for overweight and obesity were estimated using the lowest level of each organochlorine as the reference (Orsini and Greenland 2011). ORs for overweight and obesity were also estimated per interquartile range (IQR) increase in organochlorine levels (micrograms per liter). All models were weighted by the inverse of the sampling probabilities to account for the sampling design used to select the subset of CPP with organochlorine measures (Zhou et al. 2007). All analyses were conducted using STATA (release 12.1; StataCorp, College Station, TX, USA).

We used directed acyclic graphs (DAGs) to choose potential confounders for model adjustment (Greenland et al. 1999; Textor et al. 2011). The set of variables selected for adjustment were maternal race, education, socioeconomic index, prepregnancy BMI, smoking during pregnancy, and child’s birth order. This list of variables plus study center (12 strata), child’s sex, serum levels of triglycerides and total cholesterol were the covariates selected *a priori* for model adjustment. Another set of potential confounders was also assessed using the change in estimate method (i.e., change in OR ≥ 10%), starting with all variables in the models with deletion of one by one in a stepwise manner (Greenland 1989). None of the additionally tested variables (i.e., maternal age, pregnancy weight gain, weeks of gestation at enrollment, and child’s age) were selected with this strategy.

Potential interactions of the exposure with breastfeeding (child was breastfed at the nursery ≥ 1 days: no, yes), maternal smoking (no, yes), child’s sex (male, female), child born small for gestational age [SGA, defined as a birth weight for gestational age below the 10th percentile (no, yes)], and preterm birth [child born before 37 weeks of gestation (no, yes)] were examined by introducing cross-product terms for these variables [e.g., organochlorine (micrograms per liter) × breastfeeding (no, yes)] into the regression models. All interactions were tested in the context of linear regression with BMI as the outcome because of limited statistical power when modeling childhood overweight or obesity. Interactions with a *p*-value for the cross-term product ≤ 0.20 were further assessed with stratified analyses.

The following set of additional models was run in sensitivity analyses only. We estimated adjusted ORs for overweight and obesity using categories of exposure; the cut points to define categories were selected based on quantiles of distribution specific to each organochlorine, which aimed to keep observations below the LOD as the lowest category; for organochlorines with > 22% values below the LOD (i.e., *trans*-nonachlor and oxychlordane) we used tertiles and for the others we used quintiles. For each analysis, a 1-df (degrees of freedom) trend test was obtained, based on assigning to each subject an exposure value equal to the median organochlorine level among subjects in their respective organochlorine category. Overweight and obesity were also modeled using organochlorine levels expressed per lipid basis (nanograms per gram lipids) as the exposure; in that case, the regression models did not include triglycerides or total cholesterol as covariates. Child’s BMI as an outcome was also assessed in relation to organochlorine exposure by fitting linear regression models that were additionally adjusted for child’s exact age when the anthropometric measurements were taken. In addition, the analysis was restricted to the children selected at random (i.e., from the subset of the CPP cohort with organochlorine measurements), to those with organochlorine levels above the LOD, to non-breastfed children, and finally to those without intrauterine growth restriction who were also born at term. We also fitted models with additional adjustment for breastfeeding, SGA, and preterm birth. We performed multiple imputation by chained equations (van Buuren et al. 1999) for covariates with missing data, and the analyses were repeated using the imputed data (see Supplemental Material, Multiple Imputation).

## Results

The mean (± SD) age of the children in the study was 7.1 ± 0.2 years). The prevalence of childhood overweight (excluding obesity) was 8.6% and of obesity was 3.5%. Higher prevalence of overweight and obesity was observed among children whose mothers were overweight or obese before pregnancy (Table 1). The prevalence of obesity was slightly higher among children of white women than African-American women.

**Table 1:** Characteristics of the mothers and children according to child’s BMI^*a*^ at 7 years of age in the CPP, 1959–1965.

Characteristics	Normal BMI		Overweight		Obese		*p*-Value^*c*^
	*n*	Percent^*b*^ or median (IQR)	*n*	Percent^*b*^ or median (IQR)	*n*	Percent^*b*^ or median (IQR)	
All	1,683	87.8	165	8.6	68	3.5
Mother
Age at recruitment (years)	1,683	23.0 (8.0)	165	23.0 (10.0)	68	23.0 (9.0)	0.27
Race							< 0.01
White	725	85.3	90	10.6	35	4.1
African American	904	90.3	70	7.0	27	2.7
Other	54	83.1	5	7.7	6	9.2
Education							0.06
< High school	972	87.6	89	8.0	48	4.3
≥ High school	711	88.1	76	9.4	20	2.5
Socioeconomic index							0.03
≤ 3.0	468	90.5	33	6.4	16	3.1
3.1–6.0	776	86.3	82	9.1	41	4.6
> 6.0	439	87.8	50	10.0	11	2.2
Smoking status (cigarettes/day)							0.66
Nonsmoker	932	88.2	93	8.8	32	3.0
Smoke ≤ 10	469	88.0	43	8.1	21	3.9
Smoke > 10	282	86.5	29	8.9	15	4.6
Prepregnancy BMI (kg/m^2^)							< 0.01
< 25.0	1,335	89.8	110	7.4	42	2.8
25.0–29.9	243	80.7	40	13.3	18	6.0
≥ 30.0	105	82.0	15	11.7	8	6.3
Pregnancy weight gain (kg)	1,683	10.4 (5.4)	165	10.8 (5.9)	68	11.7 (5.2)	0.01
Children
Sex							0.01
Male	1,042	88.4	107	9.1	30	2.5
Female	641	87.0	58	7.9	38	5.2
Live birth order							0.09
First	502	85.4	57	9.7	29	4.9
Second	361	87.2	39	9.4	14	3.4
≥ Third	820	89.7	69	7.5	25	2.7
Breastfeeding ≥ 1 days							0.17
No	1,300	88.1	119	8.1	56	3.8
Yes	258	86.3	35	11.7	6	2.0
Missing	125	88.0	11	7.7	6	4.3
Birth length (cm)	1,664	50.0 (4.0)	165	50.0 (4.0)	67	50.0 (3.0)	0.27
Birth weight (g)	1,682	3,203 (681)	165	3,317 (709)	68	3,161 (751)	< 0.01
Weight (kg)^*d*^	1,683	22.7 (3.7)	165	28.5 (5.0)	68	35.3 (6.7)	< 0.01
Height (cm)^*d*^	1,683	121.0 (7.0)	165	123.0 (9.0)	68	125.0 (7.5)	< 0.01
BMI (kg/m^2^)^*d*^	1,683	15.5 (1.7)	165	18.7 (1.0)	68	22.3 (3.1)	< 0.01
^***a***^Categories defined by age- and sex-specific cut-off points for BMI from the IOTF. ^***b***^Some row percentages do not add up 100% due to rounding. ^***c***^*p*-Values testing differences in percentages (Pearson’s chi-square or Fisher’s exact test) or medians (Kruskal–Wallis) across the three groups defined by child’s BMI. ^***d***^At the 7-year examination.							

**Figure 1:**
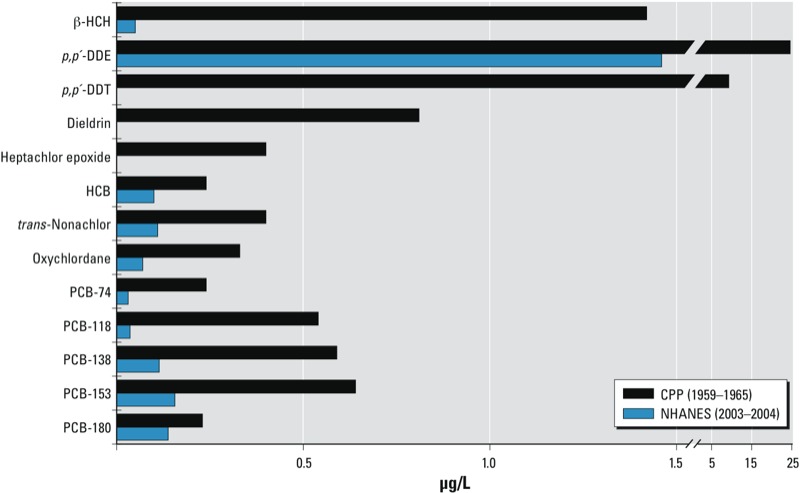
Median levels of organochlorine chemicals measured in adult serum from the NHANES compared with women from the CPP. The NHANES 50th percentile for dieldrin, heptachlor epoxide, and *p,p’*‑DDT was below the LOD (< 0.01 μg/L).

As expected, those organochlorines with higher median concentrations tended to have a greater proportion of measurements ≥ LOD (Table 2); the Spearman correlation coefficient between median concentration and proportion ≥ LOD was 0.99 (calculation based on 19 organochlorines, including specific PCB congeners). The between-assay coefficient of variation (CV) was < 25% for most of the chemicals, except for HCH (CV, 59%). Compared with adults ≥ 20 years of age from the U.S. National Health and Nutrition Examination Survey (NHANES 2003–2004), women from the CPP had higher levels of exposure to all organochlorines (Figure 1) (CDC 2009). NHANES data showed nondetectable levels of dieldrin, heptachlor epoxide, and *p,p´*-DDT in 50% of the U.S. adult population; median levels of *p,p´*-DDE were approximately 17 times higher in the CPP.

**Table 2:** Third-trimester serum levels of organochlorines among women from the CPP, 1959–1965.

Organochlorines	*n*	CV (%)	LOD (μg/L)	< LOD (%)	Selected percentiles (μg/L)			
					25th	50th	75th	95th
β-HCH	1,899	18.6	0.23	0.2	1.01	1.42	2.12	5.25
*p,p’*-DDE	1,833	19.2	0.61	0.0	16.93	24.59	36.35	69.63
*p,p’*-DDT	1,903	22.1	0.66	0.1	6.46	9.33	14.16	26.57
Dieldrin	1,807	19.9	0.23	1.1	0.60	0.81	1.09	1.81
Heptachlor epoxide	1,859	18.6	0.21	19.7	0.24	0.40	0.74	1.53
HCB	1,881	59.3	0.08	21.3	0.12	0.24	0.35	0.82
*trans*-Nonachlor	1,911	19.7	0.28	28.4	0.26	0.40	0.58	0.91
Oxychlordane	1,809	24.6	0.20	29.8	0.16	0.33	0.53	0.95
Total PCBs^*a*^	1,915	18.7	0.20–0.33	5.2–98.4	1.93	2.74	3.92	6.74
CV, coefficient of variation. ^***a***^Congener-specific LOD (μg/L): 74 and 203, 0.20; 105 and 194, 0.21; 170, 0.22; 180, 0.23; 118 and 138, 0.25; 28 and 52, 0.27; 153, 0.33. The proportion below LOD was, for 138, 5.2%; 153, 8.7%; 118, 10.2%; 74, 36.8%; 180, 49.5%; and for the other congeners, ≥ 76.8%.								

**Figure 2:**
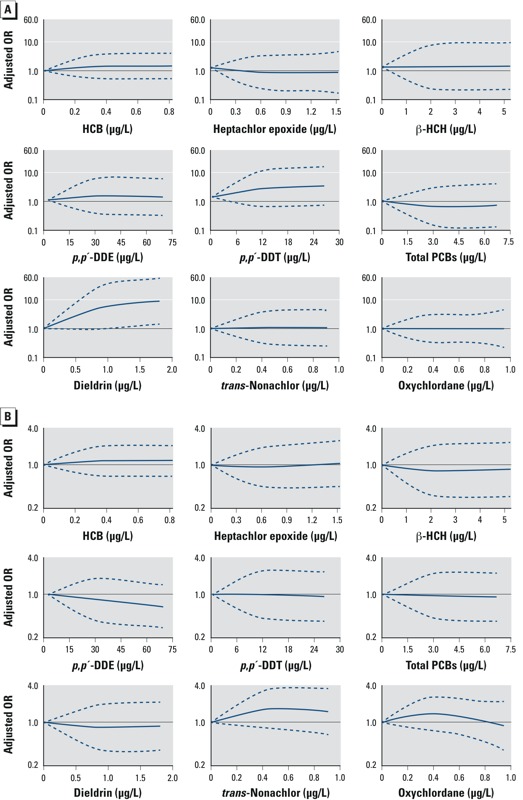
Adjusted ORs for childhood obesity (*A*) and overweight including obesity (*B*) across levels of prenatal exposure to each individual organochlorine. ORs were estimated from restricted cubic splines with three knots, the reference is the lowest level of each organochlorine. The *x*-axes were truncated at the 95th percentile of the distribution for all organochlorines. Dotted lines represent the 95% CIs.

After adjusting for potential confounders, increasing levels of HCB, heptachlor epoxide, β-HCH, *p,p´*-DDE, total PCBs, *trans*-nonachlor, and oxychlordane were not associated with childhood obesity in the present study (Figure 2A). *p,p´*-DDT was associated with an increased odds of obesity, but this was not statistically significant. Increasing levels of dieldrin were associated with obesity, but the confidence intervals were particularly wide (Figure 2A). None of the organochlorines were associated with overweight (including obesity); in particular, the ORs for dieldrin were close to the null (Figure 2B). An IQR increase in levels of HCB, heptachlor epoxide, β-HCH, *p,p´*-DDE, *p,p´*-DDT, total PCBs, *trans*-nonachlor, and oxychlordane was not associated with overweight, obesity, or BMI; dieldrin was associated with obesity [adjusted OR = 1.32; 95% CI: 1.01, 1.73] but not with overweight or BMI (Table 3). Compared with the lowest quintile of dieldrin exposure, children from higher quintiles had higher odds of obesity (*p*-trend = 0.08); however, given the small number of children classified as obese (3.5%), the estimates were imprecise as shown by the wide confidence intervals (Table 4). Similar to the results from the restricted cubic splines, no association between quintiles of dieldrin exposure and overweight or BMI were observed.

**Table 3:** Associations between maternal exposure to persistent organochlorines (per IQR increase) and offspring body size in the CPP, 1959–1965.

Chemicals (μg/L)	IQR	Overweight^*a*^	Obese	BMI (kg/m^2^)
		OR^*b*^ (95% CI)	OR^*b*^ (95% CI)	β^*b,c*^ (95% CI)
β-HCH	1.11	1.00 (0.87, 1.16)	1.03 (0.84, 1.24)	0.01 (–0.06, 0.09)
*p,p’*-DDE	19.42	0.88 (0.72, 1.07)	1.00 (0.75, 1.32)	–0.03 (–0.18, 0.11)
*p,p’*-DDT	7.70	0.97 (0.79, 1.19)	1.16 (0.88, 1.54)	0.02 (–0.12, 0.16)
Dieldrin	0.49	1.00 (0.83, 1.22)	1.32 (1.01, 1.73)	0.09 (–0.12, 0.29)
Heptachlor epoxide	0.50	1.06 (0.82, 1.36)	0.96 (0.48, 1.93)	0.00 (–0.18, 0.18)
HCB	0.23	1.00 (0.98, 1.03)	1.02 (0.99, 1.05)	0.02 (–0.02, 0.07)
*trans*-Nonachlor	0.32	1.05 (0.82, 1.34)	1.00 (0.67, 1.51)	0.01 (–0.15, 0.17)
Oxychlordane	0.37	0.92 (0.67, 1.26)	1.00 (0.53, 1.90)	–0.01 (–0.20, 0.17)
Total PCBs	1.99	0.97 (0.80, 1.18)	1.01 (0.71, 1.44)	0.01 (–0.12, 0.14)
^***a***^Includes overweight and obese. ^***b***^Adjusted for total cholesterol, triglycerides, study center, mother’s race, socio­economic index, education, smoking during pregnancy, prepregnancy BMI, and child’s sex and birth order. ^***c***^Additionally adjusted for child’s exact age at anthropometric measurements.				

**Table 4:** Associations between maternal exposure to dieldrin and offspring’s body size in the CPP, 1959–1965.

Dieldrin (μg/L)^*a*^	Overweight^*b*^		Obese		BMI (kg/m^2^)
	*n*	OR (95% CI)^*c*^	*n*	OR (95% CI)^*c*^	β (95% CI)^*c,d*^
< 0.57	50	1.00	8	1.00	0.00
0.57–0.72	37	0.78 (0.44, 1.39)	8	1.49 (0.46, 4.85)	0.07 (–0.23, 0.38)
0.73–0.91	46	0.84 (0.48, 1.48)	12	1.39 (0.47, 4.15)	0.12 (–0.16, 0.41)
0.92–1.18	41	0.83 (0.46, 1.48)	20	3.62 (1.25, 10.46)	0.22 (–0.15, 0.59)
> 1.18	48	0.79 (0.44, 1.43)	16	2.31 (0.76, 7.09)	0.13 (–0.24, 0.50)
*p*-Trend^*e*^		0.61		0.08	0.50
^***a***^Quintiles. ^***b***^Includes overweight and obese. ^***c***^Adjusted for total cholesterol, triglycerides, study center, mother’s race, socioeconomic index, education, smoking during pregnancy, prepregnancy BMI, and child’s sex and birth order. ^***d***^Additionally adjusted for child’s exact age at anthropometric measurements. ^***e***^Trend test from a model that included quintiles of dieldrin as an ordinal variable, median level of each quintile defined each category.					

Potential interactions (*p*-interaction ≤ 0.20) were observed for maternal smoking and total PCBs; for child’s sex and *p,p´*-DDT, dieldrin, heptachlor epoxide, and oxychlordane; for breastfeeding and β-HCH, *p,p´*-DDE, *p,p´*-DDT, dieldrin, and HCB; and for children born SGA with β-HCH and HCB. After stratification, however, there was little evidence of effect modification (see Supplemental Material, Tables S1–S4). Briefly, PCBs (per interquartile increase) were associated with higher child’s BMI among smoking mothers and lower child’s BMI among nonsmoking mothers (see Supplemental Material, Table S1). Increasing levels of *p,p´*-DDT, dieldrin, heptachlor epoxide, and oxychlordane tended to be associated with higher BMI among boys but lower BMI among girls (see Supplemental Material, Table S2). We were unable to stratify on breastfeeding (most children were not breastfed) and SGA because of the small numbers. However, estimates among non-breastfed children and among those with a birth weight > 10th percentile for gestational week were comparable to those in Table 3 (see Supplemental Material, Table S3, S4).

The results did not change materially when the models were refitted using categories of exposure (*p*-values for trend test were all ≥ 0.24) or when organochlorines were expressed per lipid basis (nanograms per gram lipids) (data not shown); and as before, dieldrin was associated with obesity [adjusted OR = 1.42; 95% CI: 1.01, 1.85 (per IQR increase, 64 ng/g lipid)] but not with overweight or BMI. After restricting the analyses to the group of children selected at random (i.e., from the subset of CPP cohort with organochlorine measurements) or to those with organochlorine levels above the LOD, results were consistent with those observed in Table 3 (data not shown). Among children from the random sample, the adjusted OR for obesity per interquartile increase in dieldrin was 1.21 (95% CI: 0.91, 1.61). After excluding children born preterm or SGA, some of the ORs for obesity changed by > 10% (e.g., oxychlordane: OR = 1.31; 95% CI: 0.69, 2.49; see Supplemental Material, Table S5], however, the estimates were imprecise and the association with dieldrin remained. Restricting the analysis to non-breastfeed children (the majority; only 299 children were breastfed) showed results comparable to those from Table 3 (see Supplemental Material, Table S6). The results did not materially change from those in Table 3 after additional adjustment for potentially intermediate variables (i.e., SGA, premature birth, and breastfeeding) (data not shown). The results from the multiple imputation analyses showed that the ORs for obesity were reduced by 5% when dieldrin was modeled as continuous (per IQR increase) and by 30–37% when using quintiles; otherwise, they were comparable to those based on complete data (data not shown).

## Discussion

Overall, prenatal exposure to persistent organochlorines was not associated with obesity, overweight, or BMI among children from the CPP. Increasing levels of dieldrin exposure, however, showed an association with childhood obesity, but it was not associated with the other two outcomes (i.e., overweight or BMI). This finding was consistent among children in the random sample; however, in that sample the adjusted OR for obesity did not reach statistical significance, likely due to the smaller number of obese children among this subset. Nevertheless, a chance finding cannot be ruled out given the number of exposures (*n* = 9) and outcomes (*n* = 3) assessed in the present study (for the main analyses that includes the assessment of interactions, ~ 81 models were fitted).

We are not aware of any previous study of the potential role of prenatal exposure to dieldrin in the development of obesity in humans. However, acute aldrin exposure in pregnant mice resulted in decreased body weight of the offspring [Agency for Toxic Substances and Disease Registry (ATSDR) 2002]. [Aldrin is converted to dieldrin *in vivo* (ATSDR 2002).] Recent data show low levels of serum dieldrin (75th percentile, 0.06 μg/L) among the U.S. population compared with the CPP participants (75th percentile, 1.09 μg/L) (CDC 2009).

A summary of prior studies assessing prenatal exposure to β-HCH, *p,p´*-DDE, *p,p´*-DDT, HCB, and PCBs in relation to body weight or BMI is presented in Supplemental Material, Table S7. Similar to previous studies conducted among subjects who experienced relatively high exposure, prenatal exposure to *p,p´*-DDE was not associated with BMI (Cupul-Uicab et al. 2010; Gladen et al. 2004) (see Supplemental Material, Table S7). Earlier studies that reported an association between DDE exposure and higher BMI during infancy and childhood (Mendez et al. 2011; Valvi et al. 2012; Verhulst et al. 2009) had median levels of *p,p´*-DDE that were lower than or close to the lowest level of exposure found in the CPP participants (lowest value of *p,p´*-DDE in the CPP, 0.34 μg/g lipid). In addition, these non-null findings tended to be limited to certain subgroups (i.e., higher BMI with DDE exposure among children whose mothers smoked ever or among children whose mothers had a normal prepregnancy BMI) (Mendez et al. 2011; Verhulst et al. 2009). In the present study, however, DDE did not show an interaction with maternal smoking or prepregnancy BMI.

Similar to our results, two previous studies that assessed prenatal exposure to *p,p´*-DDT in relation to BMI also reported null findings among infants and youth (Cupul-Uicab et al. 2010; Gladen et al. 2004) (see Supplemental Material, Table S7).

An association between prenatal exposure to HCB and BMI or obesity was not evident in the present study. The previous study that supported such an association was based on a population with levels of HCB (measured in cord blood) higher than those found in the CPP (Smink et al. 2008) (see Supplemental Material, Table S7).

As observed in the present study, null associations between prenatal PCBs exposure and subsequent weight or BMI (i.e., infants and adults) have been previously reported among populations with PCBs levels that are higher or lower than or similar to those found in the CPP participants (Jackson et al. 2010; Karmaus et al. 2009; Patandin et al. 1998) (see Supplemental Material, Table S7). However, our results differ from those of earlier studies that have linked higher levels of PCBs exposure with decreased weight among children and youth, which included populations with PCBs levels that were similar to or higher than the CPP (Blanck et al. 2002; Jacobson et al. 1990; Lamb et al. 2006).

As noted previously with DDE, prenatal exposure to DDT and PCBs was associated with overweight or high BMI only in studies that included populations with low levels of exposure (see Supplemental Material, Table S7), unlike the CPP. Potential effect modification of DDE and PCBs by sex, reported by earlier studies (Gladen et al. 2000; Hertz-Picciotto et al. 2005; Lamb et al. 2006), was not supported in the CPP (see Supplemental Material, Table S2, S7).

Overall, prior studies evaluating developmental exposure to persistent organochlorines in relation to body size have provided little support for the hypothesis that higher levels of exposure are linked to obesity; yet with the available evidence, an association cannot be ruled out (see Supplemental Material, Table S7). Conflicting findings across studies might be explained by methodological variations and by particular characteristics of the studied populations. However, it is also plausible that prenatal exposure to these chemicals at low levels, but not at higher levels, may promote adiposity (Grun and Blumberg 2009).

The mechanism(s) by which prenatal exposure to persistent organochlorine chemicals might be related to increased body fatness are not clear. Potential pathways might entail alterations of the hormones involved in growth regulation and adipogenesis (i.e., thyroid, steroids, and growth hormone) (Cocchi et al. 2009; Diamanti-Kandarakis et al. 2009; Garten et al. 2012; Grun and Blumberg 2009) or of regulation of behavior by the central nervous system (ATSDR 2002).

Compared with previous studies evaluating prenatal exposure to persistent organochlorines in relation to body weight or BMI, the present analysis included by far the largest number of subjects (see Supplemental Material, Table S7). Although some of the CPP children included in our study were selected based on sex-specific birth defects or their performance on various neurodevelopmental tests, the findings from the analysis that included all subjects (which accounted for selection design) were maintained when the analyses were restricted to children selected at random. Whereas the selection status of the children may be associated with some of the organochlorines, selection status was not associated with obesity, overweight, or BMI. A serious selection bias due to the inclusion of children with sex-specific birth defects or neurodevelopmental delays was therefore unlikely.

In the present study, levels of all organochlorines were much higher than those reported recently for the U.S. population (Figure 1). CPP participants experienced particularly high exposure to DDT and its main breakdown product, *p,p´*-DDE. Few people with very low levels of *p,p´*-DDE were in the CPP, thus comparing results to studies with statistically significant findings (Mendez et al. 2011; Verhulst et al. 2009) is not straightforward.

Compared with children followed to 7 years of age, those lost to follow-up had slightly higher median levels of *p,p´*-DDE (25.7 μg/L), dieldrin (0.87 μg/L), and *trans*-nonachlor (0.44 μg/L), but they had slightly lower levels of heptachlor epoxide (0.37 µg/L), HCB (0.21 μg/L), and PCBs (2.55 μg/L). This pattern shows that non-followed children did not consistently have higher levels of exposure; furthermore, given the small differences in exposure between children who were or were not followed, selection bias seems unlikely.

Children from the CPP were prospectively followed, and their height and weight were measured by trained personnel who did not know the child’s exposure status; thus, differential misclassification of the outcomes was unlikely. Using obesity as an outcome may be a better surrogate measure of adiposity than BMI because high BMI among relatively thin children may not reflect excess body fatness (Freedman and Sherry 2009). Because there are no unified criteria to classify children as overweight or obese, these two outcomes were defined using the IOTF criteria (age- and sex-specific cut points) that allow comparison of the prevalence across countries (Cole et al. 2000). Among 6- to 8-year-olds, the IOTF cut points to classify overweight children have high sensitivity (> 83%) and specificity (> 91%), but the cut points to classify obese children have low sensitivity (68%) and high specificity (> 98%) compared with percentage of body fat (Zimmermann et al. 2004). Using percentage of body fat as the gold standard, the IOTF criteria to define overweight and obesity in children 6–8 years of age showed lower sensitivity but higher specificity than the CDC criteria (Zimmermann et al. 2004).

Compared with recent data from U.S. children (Orsi et al. 2011), the prevalence of overweight and obesity among children in the CPP was low, which decreased the statistical power of the study, especially to assess interactions when modeling these outcomes. All interactions were tested using BMI as the outcome, as was done in previous studies with smaller samples sizes; however, previously reported interactions (i.e., exposure with maternal smoking and child’s sex) were not replicated in the present study. Thus, it is not clear whether the exposure has differential effects on child’s BMI as previously suggested. Our data did not support interactions between prenatal exposure to organochlorines and fetal growth restriction (i.e., SGA) or prematurity; however, among children without fetal growth restriction who were born at term, organochlorine exposure was not associated with body size at 7 years, except for dieldrin and obesity, as before (see Supplemental Material, Table S5).

Except for HCB, the between-assay CV was relatively low for all organochlorines measured in the present study. Although for some chemicals the percentage of samples below the LOD was 20–30%, our results were unchanged when the analysis was restricted to the sample with levels above the LOD.

## Conclusions

In the present study, which included a relatively large sample of children with comparatively high prenatal exposure to persistent organochlorines, no clear associations emerged between exposure and obesity or BMI. The suggestive association between dieldrin and childhood obesity was perhaps a chance finding given the number of analyses we performed. However, the present data do not refute a potential role of prenatal exposure to persistent organochlorines in the development of obesity. Low levels of exposure to persistent organochlorines in the prenatal period could conceivably promote obesity, but overall these data provide little support for an association at higher levels.

## Supplemental Material

(401 KB) PDF
