# Supplementary material for: Prenatal Exposure to Persistent Organochlorines and Childhood Obesity in the U.S. Collaborative Perinatal Project
Source: Environ Health Perspect. 2013 Jun 25;121(9):1103–9. doi: 10.1289/ehp.1205901 (PMC3764072; doi:10.1289/ehp.1205901)
Supplement: (401 KB) PDF [file ehp.1205901.s001.pdf]

Supplemental Material

Prenatal Exposure to Persistent Organochlorines and Childhood Obesity in the U.S. Collaborative Perinatal Project

Lea A. Cupul-Uicab, Mark A. Klebanoff, John W. Brock, Matthew P. Longnecker

Corresponding author:

Center for Population Health Research, National Institute of Public Health, Avenida Universidad 655, Cuernavaca, Morelos 62100, Mexico; e-mail: lea.cupul@insp.mx; cupuluicabl@niehs.nih.gov; phone +52(777)329-3000 Ext. 3204

Contents

Multiple imputation 3

Supplemental Material, Table S1. Association of prenatal exposure to PCBs and BMI (kg/m2) at age 7 years in the US CPP, stratified by maternal smoking 4

Supplemental Material, Table S2. Associations of prenatal exposure to organochlorines and BMI at age 7 years in the US CPP, stratified by child's sex 5

Supplemental Material, Table S3. Associations of prenatal exposure to organochlorines and BMI at age 7 years among non-breastfed children from the US CPP...............6

Supplemental Material, Table S4. Associations of prenatal exposure to organochlorines and BMI at age 7 years among children with birth weight >10th percentile for gestational age in the US CPP...7

Supplemental Material, Table S5. Associations between maternal exposure to persistent organochlorines (per interquartile increase) and offspring's body size in the CPP, 1959-1965. Excluding preterm born and SGA children 8

Supplemental Material, Table S6. Associations between maternal exposure to persistent organochlorines (per interquartile increase) and offspring's body size in the CPP, 1959-1965. Restricted to non-breastfed children......................................9

Supplemental Material, Table S7. Summary of studies on body size in relation to prenatal exposure to persistent organochlorines 10

References 14

Multiple imputation

Multiple imputation by chained equations (MICE) (van Buuren et al. 1999) were performed to impute values for any covariate with missing data [i.e. maternal pre-pregnancy BMI (n=144), smoking (n=11), pregnancy weight gain (n=8), and birth order (n=2)]. For the imputation procedure we included all maternal and child characteristic listed in Table 1 (except maternal and child BMI), maternal weight before pregnancy and height, week of gestation at enrollment in the CPP, child's exact age at the 7 year examination, all organochlorines, total cholesterol and triglycerides, selection status (i.e., sex-specific birth defects, random sample), and study center. A total of 10 imputed datasets were generated using 20 cycles per imputation (van Buuren et al. 1999); the main analyses were repeated using the imputed data.

Supplemental Material, Table S1. Association of prenatal exposure to PCBs and BMI (kg/m2) at age 7 years in the US CPP, stratified by maternal smoking

	Mother smoked during pregnancy				
	No		Yes		
Chemical (μg/L)	n	β[a] (95%CI)	n	β[a] (95%CI)	*p*-interaction*
PCBs	1057	-0.09 (-0.23, 0.04)	858	0.15 (-0.09, 0.40)	0.09

BMI, body mass index; PCBs, polychlorinated biphenyls

[a] Interquartile increase, 1.99 μg/L. Adjusted for total cholesterol, triglycerides, study center, mother's race, socioeconomic index, pre-pregnancy BMI, education, child's gender, child's exact age at anthropometric measurements, and birth order

*Interaction between PCBs exposure and maternal smoking during pregnancy

Supplemental Material, Table S2. Associations of prenatal exposure to organochlorines and BMI at age 7 years in the US CPP, stratified by child's sex

		Boys		Girls		
Chemicals (μg/L)	IQR	n	β[a] (95%CI)	n	β[a] (95%CI)	*p*-interaction*
p,p'-DDT	7.70	1171	0.08 (-0.11, 0.26)	732	-0.02 (-0.23, 0.18)	0.20
Dieldrin	0.49	1117	0.29 (-0.02, 0.60)	690	-0.09 (-0.20, 0.03)	0.05
HE	0.50	1151	0.10 (-0.13, 0.33)	708	-0.16 (-0.39, 0.08)	0.01
Oxychlordane	0.37	1114	0.03 (-0.22, 0.27)	695	-0.10 (-0.38, 0.19)	0.03

BMI, body mass index; HE, heptachlor epoxide; IQR, interquartile range

[a] Increase per interquartile range. Adjusted for total cholesterol, triglycerides, study center, mother's race, socioeconomic index, pre-pregnancy BMI, education, smoking during pregnancy, child's exact age at anthropometric measurements, and birth order

*Interaction between organochlorine exposure and child's sex

Supplemental Material, Table S3. Associations of prenatal exposure to organochlorines and BMI at age 7 years among non-breastfed children from the US CPP

Chemicals (μg/L)	IQR	n	β[a] (95%CI)
β-HCH	1.11	1461	0.02 (-0.07, 0.11)
p,p′-DDE	19.42	1408	-0.01 (-0.19, 0.16)
p,p′-DDT	7.70	1464	0.03 (-0.13, 0.19)
Dieldrin	0.49	1398	0.11 (-0.13, 0.36)
HCB	0.23	1446	0.04 (-0.03, 0.12)

β-HCH, β-hexachlorocyclohexane; BMI, body mass index; HCB, hexachlorobenzene; IQR, interquartile range

[a] Increase per interquartile range. Adjusted for total cholesterol, triglycerides, study center, mother's race, socioeconomic index, pre-pregnancy BMI, education, smoking during pregnancy, child's sex, child's exact age at anthropometric measurements, and birth order

Supplemental Material, Table S4. Associations of prenatal exposure to organochlorines and BMI at age 7 years among children with birth weight >10th percentile for gestational age in the US CPP

Chemicals (μg/L)	IQR	n	β[a] (95%CI)
β-HCH	1.11	1698	0.00 (-0.05, 0.05)
HCB	0.23	1684	0.02 (-0.02, 0.07)

β-HCH, β-hexachlorocyclohexane; BMI, body mass index; HCB, hexachlorobenzene; IQR, interquartile range

[a] Increase per interquartile range. Adjusted for total cholesterol, triglycerides, study center, mother's race, socioeconomic index, pre-pregnancy BMI, education, smoking during pregnancy, child's sex, child's exact age at anthropometric measurements, and birth order

Supplemental Material, Table S5. Associations between maternal exposure to persistent organochlorines (per interquartile increase) and offspring's body size in the CPP, 1959-1965. Excluding preterm born and SGA children

Chemicals (µg/L)	N	IQR	Overweight[a] OR[b] (95% CI)	Obese OR[b] (95% CI)	BMI (kg/m^2) $\beta^{b,c}$ (95% CI)
β-HCH	1476	1.11	0.96 (0.82, 1.14)	0.88 (0.71, 1.09)	-0.02 (-0.07, 0.04)
p,p′-DDE	1436	19.42	0.89 (0.70, 1.13)	0.93 (0.65, 1.34)	-0.04 (-0.22, 0.14)
p,p′-DDT	1480	7.70	1.04 (0.80, 1.35)	1.19 (0.81, 1.75)	0.05 (-0.13, 0.22)
Dieldrin	1401	0.49	1.00 (0.79, 1.26)	1.38 (1.03, 1.85)	0.09 (-0.16, 0.33)
HE	1456	0.50	1.03 (0.77, 1.38)	1.08 (0.58, 2.01)	-0.02 (-0.23, 0.19)
HCB	1463	0.23	1.00 (0.98, 1.03)	1.02 (0.99, 1.06)	0.02 (-0.02, 0.07)
t-Nonachlor	1488	0.32	1.14 (0.84, 1.55)	1.04 (0.59, 1.85)	0.03 (-0.18, 0.23)
Oxychlordane	1411	0.37	1.05 (0.74, 1.50)	1.31 (0.69, 2.49)	0.05 (-0.17, 0.28)
PCBs	1491	1.99	0.98 (0.79, 1.23)	0.97 (0.61, 1.54)	0.01 (-0.14, 0.16)

β-HCH, β-hexachlorocyclohexane; BMI, body mass index; HCB, hexachlorobenzene; HE, heptachlor epoxide; IQR, interquartile range; PCBs, polychlorinated biphenyls

[a] Includes overweight and obese

[b] Adjusted for total cholesterol, triglycerides, study center, mother's race, socioeconomic index, education, smoking during pregnancy, pre-pregnancy BMI, child's gender and birth order

[c] Additionally adjusted for child's exact age at anthropometric measurements

Supplemental Material, Table S6. Associations between maternal exposure to persistent organochlorines (per interquartile increase) and offspring's body size in the CPP, 1959-1965. Restricted to non-breastfed children

Chemicals (µg/L)	N	IQR	Overweight[a] OR[b] (95% CI)	Obese OR[b] (95% CI)	BMI (kg/m²) β[b,c] (95% CI)
β-HCH	1461	1.11	1.04 (0.89, 1.220	1.05 (0.84, 1.31)	0.02 (-0.07, 0.11)
p,p′-DDE	1408	19.42	0.94 (0.77, 1.16)	1.04 (0.78, 1.39)	-0.01 (-0.19, 0.16)
p,p′-DDT	1464	7.70	1.04 (0.84, 1.28)	1.21 (0.91, 1.60)	0.03 (-0.13, 0.19)
Dieldrin	1398	0.49	1.05 (0.86, 1.28)	1.35 (1.01, 1.81)	0.11 (-0.13, 0.36)
HE	1429	0.50	1.05 (0.78, 1.43)	1.06 (0.55, 2.03)	0.01 (-0.25, 0.27)
HCB	1446	0.23	0.99 (0.91, 1.08)	1.06 (0.96, 1.16)	0.04 (-0.03, 0.12)
t-Nonachlor	1471	0.32	1.04 (0.78, 1.38)	1.08 (0.71, 1.65)	0.00 (-0.18, 0.18)
Oxychlordane	1389	0.37	0.88 (0.61, 1.28)	1.03 (0.53, 2.01)	-0.05 (-0.28, 0.18)
PCBs	1474	1.99	0.96 (0.76, 1.22)	0.97 (0.63, 1.49)	-0.01 (-0.17, 0.15)

β-HCH, β-hexachlorocyclohexane; BMI, body mass index; HCB, hexachlorobenzene; HE, heptachlor epoxide; IQR, interquartile range; PCBs, polychlorinated biphenyls

[a] Includes overweight and obese

[b] Adjusted for total cholesterol, triglycerides, study center, mother's race, socioeconomic index, education, smoking during pregnancy, pre-pregnancy BMI, child's gender and birth order

[c] Additionally adjusted for child's exact age at anthropometric measurements

Supplemental Material, Table S7. Summary of studies on body size in relation to prenatal exposure to persistent organochlorines

			Median levels (µg/g lipids)[a] and main findings									
Location (children born)	N	Outcome (age in years)	β-HCH		*p,p'*-DDE		*p,p'*-DDT		HCB		PCBs	
Michigan, US[b] (1950-1980) (Karmaus et al. 2009)	176 women	Weight, BMI (20 to 50)			0.56[c]	Statistically significant increase in endpoint					0.31[c]	Null finding
New York, US[b,d] (1959-1962) (Lamb et al. 2006)	150	Weight (4, 7, 17)									1.1	Statistically significant decrease in endpoint among girls. Null among boys
Philadelphia, US (1959-1966) (Gladen et al. 2004)	304 boys	BMI, central adiposity (10 to 20)			5.7	Null finding	1.9	Null finding				
California, US (1964-1967) (Hertz-Picciotto et al. 2005)	399	Weight (5)									0.62	NS increase in endpoint among girls. Null among boys
Michigan, US[b] (1976-1979) (Blanck et al. 2002)	305 girls	Weight (5 to 24)									0.63[c]	Statistically significant decrease in endpoint

Location (children born)	N	Outcome (age in years)	Median levels (µg/g lipids)[a] and main findings									
			β-HCH		*p,p'*-DDE		*p,p'*-DDT		HCB		PCBs	
North Carolina, US[b] (1978-1982) (Gladen et al. 2000)	594	Weight (10 to 17)			1.6	Null among girls. Statistically significant increase in endpoint among boys					1.14	NS increase in endpoint among girls. Null among boys
Michigan, US[b] (1980-1981) (Jacobson et al. 1990)	123	Weight (4)									0.96[e,f]	Statistically significant decrease in endpoint
The Netherlands[b] (1990-1992) (Patandin et al. 1998)	207	Weight change (0.3 to 3.5)									0.26	Null finding
New York State, US (1996-2002) (Jackson et al. 2010)	44	WFA, WFL *z*-score (2)									1.17	Null finding
Menorca, Spain[b] (1997-1998) (Smink et al. 2008)	405	Weight, BMI (6.5)							0.26[f]	Statistically significant increase in endpoint		
Menorca, Spain[b] (1997-1998) (Valvi et al. 2012)	344	Overweight or obesity (6.5)			0.41[f]	Statistically significant increase in endpoint in second but not third tertile	0.03[f]	NS increase in endpoint			0.29[f]	Statistically significant increase in endpoint

Location (children born)	N	Outcome (age in years)	Median levels (µg/g lipids)[a] and main findings									
			β-HCH		*p,p'*-DDE		*p,p'*-DDT		HCB		PCBs	
Morelos, Mexico (2001-2005) (Garced et al. 2012)	253	WFA, WFL, BMI-for-age *z*-scores (1)			0.71[g]	Null finding						
Chiapas, Mexico (2002-2003) (Cupul-Uicab et al. 2010)	788 boys	BMI SDS (0.4 to 3.2)			2.7	Null finding	0.3	Null finding				
Flanders, Belgium (2002-2004) (Verhulst et al. 2009)	138	BMI SDS (1 to 3)			0.21[e,f]	Statistically significant increase in endpoint with interactions by maternal smoking and child's age. Stronger associations at age 1 year and among children whose mothers ever smoked			0.03[e,f]	Null finding	0.12[e,f]	Statistically significant increase in endpoint
Catalonia, Spain[h] (2004-2006) (Mendez et al. 2011)	502	BMI-for-age *z*-score (1.2)	0.02[g]	Null finding	0.13[g]	Statistically significant increase in endpoint limited to children whose mothers had normal pre-pregnancy weight			0.02[g]	Null finding	0.04[g]	Null finding
Present study, US (1959-1965)	1,809	BMI, overweight, obesity (7)	0.18	Null finding	3.12	Null finding	1.17	Null finding	0.03	Null finding	0.34	Null finding

BMI, body mass index; β-HCH, β-hexachlorocyclohexane; HCB, hexachlorobenzene; NS, no statistically significant; PCBs, total polychlorinated biphenyls; SDS, standard deviation scores; WFA, weight-for-age; WFL, weight-for-length

[a] From maternal serum, unless otherwise noted

[b] Lipid adjusted levels were estimated assuming 8 g lipid/L in maternal serum or 2.6 g lipid/L in cord (Longnecker et al. 2003; Valvi et al. 2012)

[c] Prenatal exposure was extrapolated or estimated

[d] Included only African-American

[e] Arithmetic means

[f] Measured in cord serum or plasma

[g] Geometric means

[h] Exposure levels are from the subgroup defined as average/slow growers (levels were slightly higher among the rapid growers)

References

Blanck HM, Marcus M, Rubin C, Tolbert PE, Hertzberg VS, Henderson AK, et al. 2002. Growth in girls exposed in utero and postnatally to polybrominated biphenyls and polychlorinated biphenyls. Epidemiology 13(2):205-210.

Cupul-Uicab LA, Hernandez-Avila M, Terrazas-Medina EA, Pennell ML, Longnecker MP. 2010. Prenatal exposure to the major DDT metabolite 1,1-dichloro-2,2-bis(p-chlorophenyl)ethylene (DDE) and growth in boys from Mexico. Environ Res 110(6):595-603.

Garced S, Torres-Sanchez L, Cebrian ME, Claudio L, Lopez-Carrillo L. 2012. Prenatal dichlorodiphenyldichloroethylene (DDE) exposure and child growth during the first year of life. Environ Res 113:58-62.

Gladen BC, Klebanoff MA, Hediger ML, Katz SH, Barr DB, Davis MD, et al. 2004. Prenatal DDT exposure in relation to anthropometric and pubertal measures in adolescent males. Environ Health Perspect 112(17):1761-1767.

Gladen BC, Ragan NB, Rogan WJ. 2000. Pubertal growth and development and prenatal and lactational exposure to polychlorinated biphenyls and dichlorodiphenyl dichloroethene. J Pediatr 136(4):490-496.

Hertz-Picciotto I, Charles MJ, James RA, Keller JA, Willman E, Teplin S. 2005. In utero polychlorinated biphenyl exposures in relation to fetal and early childhood growth. Epidemiology 16(5):648-656.

Jackson LW, Lynch CD, Kostyniak PJ, McGuinness BM, Louis GM. 2010. Prenatal and postnatal exposure to polychlorinated biphenyls and child size at 24 months of age. Reprod Toxicol 29(1):25-31.

Jacobson JL, Jacobson SW, Humphrey HE. 1990. Effects of exposure to PCBs and related compounds on growth and activity in children. Neurotoxicol Teratol 12(4):319-326.

Karmaus W, Osuch JR, Eneli I, Mudd LM, Zhang J, Mikucki D, et al. 2009. Maternal levels of dichlorodiphenyl-dichloroethylene (DDE) may increase weight and body mass index in adult female offspring. Occup Environ Med 66(3):143-149.

Lamb MR, Taylor S, Liu X, Wolff MS, Borrell L, Matte TD, et al. 2006. Prenatal exposure to polychlorinated biphenyls and postnatal growth: a structural analysis. Environ Health Perspect 114(5):779-785.

Longnecker MP, Wolff MS, Gladen BC, Brock JW, Grandjean P, Jacobson JL, et al. 2003. Comparison of polychlorinated biphenyl levels across studies of human neurodevelopment. Environ Health Perspect 111(1):65-70.

Mendez MA, Garcia-Esteban R, Guxens M, Vrijheid M, Kogevinas M, Goni F, et al. 2011. Prenatal organochlorine compound exposure, rapid weight gain, and overweight in infancy. Environ Health Perspect 119(2):272-278.

Patandin S, Koopman-Esseboom C, de Ridder MA, Weisglas-Kuperus N, Sauer PJ. 1998. Effects of environmental exposure to polychlorinated biphenyls and dioxins on birth size and growth in Dutch children. Pediatr Res 44(4):538-545.

Smink A, Ribas-Fito N, Garcia R, Torrent M, Mendez MA, Grimalt JO, et al. 2008. Exposure to hexachlorobenzene during pregnancy increases the risk of overweight in children aged 6 years. Acta Paediatr 97(10):1465-1469.

Valvi D, Mendez MA, Martinez D, Grimalt JO, Torrent M, Sunyer J, et al. 2012. Prenatal concentrations of polychlorinated biphenyls, DDE, and DDT and overweight in children: a prospective birth cohort study. Environ Health Perspect 120(3):451-457.

van Buuren S, Boshuizen HC, Knook DL. 1999. Multiple imputation of missing blood pressure covariates in survival analysis. Statistics in medicine 18(6):681-694.

Verhulst SL, Nelen V, Hond ED, Koppen G, Beunckens C, Vael C, et al. 2009. Intrauterine exposure to environmental pollutants and body mass index during the first 3 years of life. Environ Health Perspect 117(1):122-126.
